# Perinatal Post‐Anoxic Spinal Cord Injury: A Barrier to Pallidal Neuromodulation? About 2 Cases

**DOI:** 10.1002/mdc3.70602

**Published:** 2026-03-20

**Authors:** Marylou Grasso, Cassandra Coelho, Emilie Chan‐Seng, Philippe Coubes, Gaëtan Poulen

**Affiliations:** ^1^ Research Unit (URCMA: Unité de Recherche sur les Comportements et Mouvements Anormaux), Centre Hospitalo‐Universitaire Gui de Chauliac Montpellier France; ^2^ Stereotactic and Functional Neurosurgery Unit, Département de neurochirurgie, Unité “Pathologie Cérébrales Résistantes,” Centre Hospitalo‐Universitaire Gui de Chauliac Montpellier France; ^3^ MMDN, University of Montpellier, EPHE, INSERM Montpellier France

**Keywords:** deep brain stimulation, imaging, perinatal hypoxic‐ischemic encephalopathy, spinal cord injury

Perinatal hypoxic–ischemic encephalopathy (HIE) is a leading cause of dystonia‐dyskinesia syndromes (DDS) through cortical‐striatal‐thalamo‐cortical network injury, resulting in persistent hyperkinetic motor disorders.^1^ Here, DDS refers to a persistent hyperkinetic phenotype dominated by dyskinesias and associated with dystonic phenomena, offering greater specificity than cerebral palsy while acknowledging areas of overlap.

Deep brain stimulation of the internal globus pallidus (GPi‐DBS) is increasingly proposed for severe, pharmacoresistant secondary dystonia, yet outcomes remain heterogeneous. Identifying predictors of DBS response is crucial to refine patient selection.

Previous studies show that sensorimotor and corticospinal pathway integrity predicts GPi‐DBS outcomes in acquired dystonia. In perinatal brain injury, preserved CMCTs were associated with better motor outcomes (*P* = 0.008), as well normal somatosensory evoked potentials (SEP) (*P* = 0.023). Combined abnormalities predicted the poorest outcomes (*P* = 0.017 and *P* = 0.002).^2^

Although perinatal hypoxia–ischemia predominantly affects the brain, spinal cord involvement has been described in both human and experimental models, including gray and white matter loss, neuronal degeneration, and corticospinal tract disruption.^3^ The impact of spinal magnetic resonance imaging (MRI) abnormalities, possibly reflecting Wallerian degeneration after perinatal injury, on DBS efficacy remains unexplored.

We report 2 patients with HIE‐related DDS who showed no clinical benefit from GPi‐DBS, both exhibiting abnormal spinal MRI and electrophysiological findings (Fig. 1).

**Fig 1:**
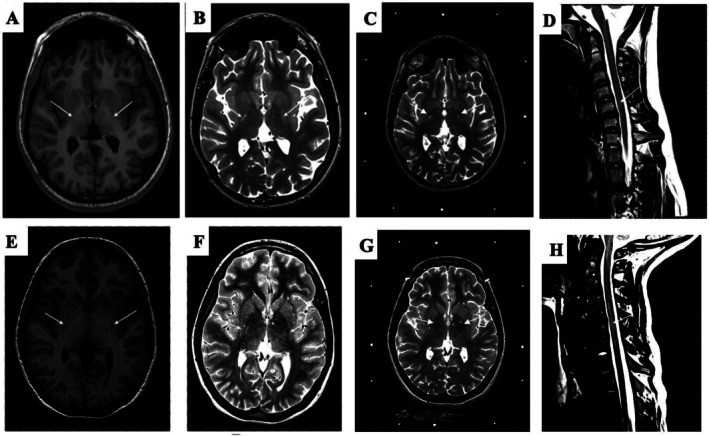
Case 1: (A) T1‐weighted images show bilateral motor putamen atrophy (white arrows), producing an “Olympic torch” appearance with a radish‐tail configuration, typically seen in perinatal anoxic brain lesions. (B) Corresponding T2‐weighted images demonstrate bilateral putaminal hyperintensities (white arrows). (C) Postoperative T2‐weighted images confirm bilateral DBS (deep brain stimulation) lead implantation in the GPi (internal globus pallidus, white arrow). (D) Cervical spinal MRI (magnetic resonance imaging) reveals centro‐medullary T2 hyperintensities (white arrow). Case 2: (E) T1‐weighted images show mild bilateral motor putamen atrophy (white arrows). (F) T2‐weighted images demonstrate bilateral motor putamen hyperintensities (white arrows). (G) Postoperative T2‐weighted images confirm bilateral DBS lead implantation in the GPi (white arrow). (H) Cervical spinal MRI reveals centro‐medullary hyperintensities on T2‐weighted images (white arrows).

## Case 1

A 21‐year‐old woman presented with generalized DDS with right predominance after perinatal anoxia, including hypertonia and dyskinesia, pyramidal signs, and spasticity. She underwent uncomplicated GPi‐DBS at 14 years with optimal lead placement. Clinical scores (Burke Fahn Marsden's dystonia rating scale ‐ motor BFMDRS‐M, 67.5; Barry‐Albright dystonia rating scale BADRS, 22) remained unchanged at 1 and 5 years. Brain MRI revealed typical anoxic changes with bilateral putaminal atrophy and hyperintensities. Spinal MRI demonstrated centromedullary T2 hyperintensities in the cervical cord. Motor evoked potentials (MEP) and SEPs were altered in all limbs.

## Case 2

A 25‐year‐old woman with perinatal injury presented with generalized dystonia and dyskinesia without spasticity, characterized by slow athetoid movements, and underwent uncomplicated GPi‐DBS at age 17, with optimal lead placement. BFMDRS‐M (31.5) and BADRS (15) scores showed no improvement at 1 and 5 years. Brain MRI showed bilateral putaminal hyperintensities and atrophy, whereas spinal MRI revealed centromedullary T2 hyperintensities without atrophy. MEPs were normal, but SEPs were abnormal in the lower limbs.

In both cases, the absence of motor improvement was associated with spinal cord abnormalities—structural (T2 hyperintensities) and/or functional (evoked potential alterations)—suggesting that spinal integrity may condition the motor response to pallidal stimulation.

Spinal involvement after perinatal anoxia is well documented histologically but rarely explored clinically.^4^, ^5^ Spinal abnormalities long after perinatal injury likely reflect secondary degeneration of motor and sensory pathways, such as Wallerian degeneration, rather than primary cord lesions, and usually occur alongside cerebral damage rather than in isolation.

Hypoxia‐induced anterior horn and corticospinal pathway injury may cause persistent hypertonia and impaired inhibitory control, whereas disrupted sensory tracts could further compromise sensorimotor integration. These mechanisms could limit the capacity of GPi‐DBS to modulate motor circuits effectively.

Our observations suggest that abnormal spinal MRI or evoked potentials might represent negative prognostic markers for GPi‐DBS efficacy in post‐anoxic dystonia. Routine spinal imaging and neurophysiological evaluation should therefore be considered before surgery to identify patients unlikely to benefit.

Further studies are warranted to determine the prevalence of spinal cord lesions after perinatal hypoxia and its correlation with DBS outcomes. Recognition of spinal lesions as potential contraindications could refine patient selection and avoid unnecessary surgical interventions in nonresponders.

## Author Roles

(1) Research project: A. Conception, B. Organization, C. Execution; (2) Analysis: A. Design, B. Execution, C. Review and critique; (3) Manuscript: A. Writing of the first draft, B. Review and critique.

M.G.: 1B, 1C, 2B, 3A

C.S.: 1C, 2C, 3B

E.C.‐S.: 1C, 2C, 3B

P.C.: 1A, 2A, 2C, 3B

G.P.: 1A, 2A, 2C, 3B

## Disclosures

**Ethical Compliance Statement:** The study was approved by the local Institutional Review Board of the University Medical Center of Montpellier. Written informed consent was obtained from all patients and/or caregivers. We confirm that we have read the journal's position on issues involved in ethical publication and affirm that this work is consistent with those guidelines.

## Data Availability

The data that support the findings of this study are available from the corresponding author upon reasonable request.
